# synphage: a pipeline for phage genome synteny graphics focused on gene conservation

**DOI:** 10.1093/bioadv/vbae126

**Published:** 2024-08-29

**Authors:** Virginie Grosboillot, Anna Dragoš

**Affiliations:** Department of Microbiology, Biotechnical Faculty, University of Ljubljana, 1000 Ljubljana, Slovenia; Department of Microbiology, Biotechnical Faculty, University of Ljubljana, 1000 Ljubljana, Slovenia

## Abstract

**Motivation:**

Visualization and comparison of genome maps of bacteriophages can be very effective, but none of the tools available on the market allow visualization of gene conservation between multiple sequences at a glance. In addition, most bioinformatic tools running locally are command line only, making them hard to setup, debug, and monitor.

**Results:**

To address these motivations, we developed synphage, an easy-to-use and intuitive tool to generate synteny diagrams from GenBank files. This software has a user-friendly interface and uses metadata to monitor the progress and success of the data transformation process. The output plot features colour-coded genes according to their degree of conservation among the group of displayed sequences. The strength of synphage lies also in its modularity and the ability to generate multiple plots with different configurations without having to re-process all the data. In conclusion, synphage reduces the bioinformatic workload of users and allows them to focus on analysis, the most impactful area of their work.

**Availability and implementation:**

The synphage tool is implemented in the Python language and is available from the GitHub repository at https://github.com/vestalisvirginis/synphage. This software is released under an Apache-2.0 licence. A PyPI synphage package is available at https://pypi.org/project/synphage/ and a containerized version is available at https://hub.docker.com/r/vestalisvirginis/synphage. Contributions to the software are welcome whether it is reporting a bug or proposing new features and the contribution guidelines are available at https://github.com/vestalisvirginis/synphage/blob/main/CONTRIBUTING.md.

## 1 Introduction

Phage genomes are characteristically mosaic and often composed of genes with different evolutionary histories (Mavrich and Hatfull 2017). Knowledge on gene conservation level and gene uniqueness within a group of related phages (e.g. single genus) allows the prediction of core structural genes required for horizontal transmission or unique accessory genes that can modulate specific host phenotypes (Owen *et al.* 2021). This facilitates the identification of potential phage recombination hotspots (De Paepe *et al.* 2014, Dragoš *et al.* 2021) or direct detection of phage recombination events detrimental to the host, such as the acquisition of genes allowing the phage to counter the host defence system (Garb *et al.* 2022). Representation of a group of related bacteriophage genomes as aligned diagrams allows clear and effective visualization of conserved versus unique genes or gene clusters.

Although several tools for phage genome visualization and synteny are already available (Cresawn *et al.* 2011, Delattre *et al.* 2016, Turner *et al.* 2018, Gilchrist and Chooi 2021), none allows users to immediately access gene conservation level or identify unique genes within a group of closely related sequences. We therefore need a flexible tool that can represent linear diagrams of several genomes, which is actively maintained and compatible with the latest Python libraries. Additionally, it must be able to run locally and not require users to upload/share data to a web server. It should also run on commodity hardware even though computing multiple files simultaneously and allow rapid visualization of gene conservation within a group of sequences.

We therefore developed synphage, a tool capable of processing multiple sequences at once. This highly modular pipeline allows the users to import local sequences but also download sequences into the pipeline, as well to perform blastn and/or blastp on the data and, offers the possibility to effortlessly generate multiple graphics from the same set of sequences for publication without the need to reprocess the data.

## 2 Description of synphage

The synphage software is implemented in Python, which makes the code accessible to a large community of users (PYPL 2024, TIOBE 2024). It relies on GenomeDiagram (Pritchard *et al.* 2006), part of the open source BioPython package (Chapman and Chang 2000, Cock *et al.* 2009) that offers multiple tools to manipulate and analyse genome sequences. It also makes use of Dagster (https://github.com/dagster-io/dagster), a powerful tool that can orchestrate pipelines and offers a web interface and, Cuallee package (Vazquez and Grosboillot 2024) a Python library used to assess data quality.

### 2.1 Input files

As input, the synphage tool requires GenBank files (.gb or .gbk) of genomes that users want to analyse.

### 2.2 File processing

**Figure 1.**
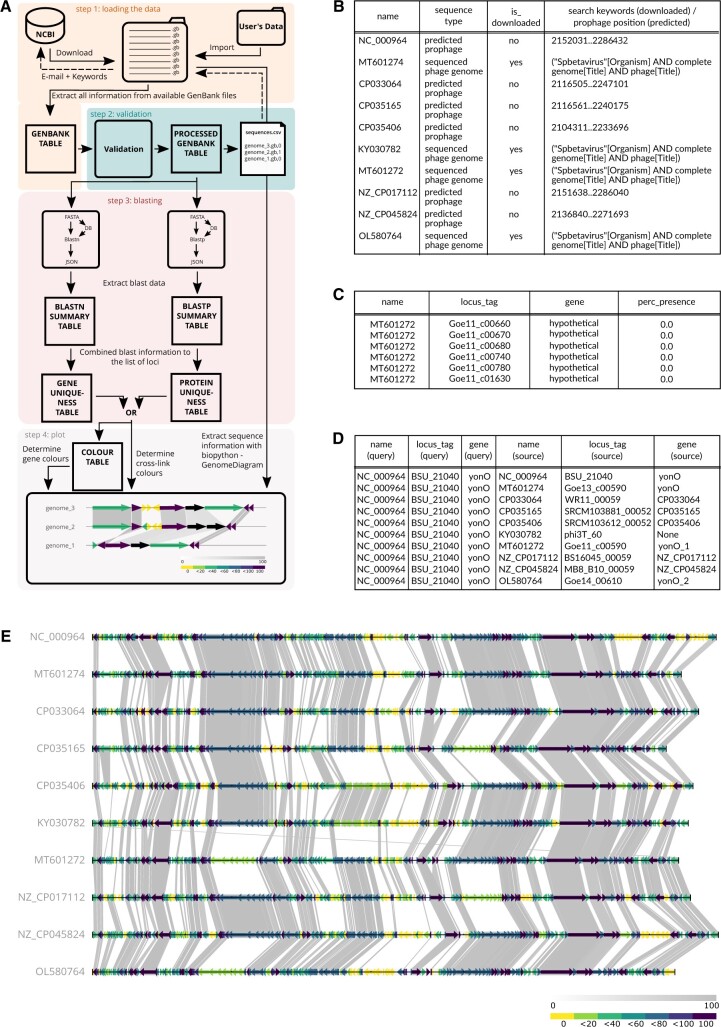
The synphage workflow and use cases. (A) Schema of the synphage workflow. (B) Origin of the data used for the example plot. (C) Colour table can be queried to retrieve unique genes by applying filters for a given sequence (e.g. MT601272). (D) Uniqueness table can be queried to obtain a list of sequences applying filters for a specific locus or gene (e.g. *yonO*) and for a selected sequence (e.g. NC_000694). (E) Synteny plot obtained for the 10 *SPbetavirus* genomes listed in B. The upper colour gradient bar corresponds to the percentage identity between two cross-linked genes. The lower colour bar represents the percentage of conservation within the displayed sequences.

The core of the software is composed of four different steps (Fig. 1A): (i) loading users’ GenBank files or downloading GenBank files from the National Center for Biotechnology Information (NCBI) database, (ii) validation of the completeness of the data, (iii) blast, and (iv) plot. After loading or downloading the GenBank files into the pipeline, the information is extracted from the files and stored in the parquet format, an optimized tabular format designed for analytic workloads. Checks are run on each dataset to assess the quality of the data to prevent downstream failures due to inconsistencies across GenBank files. The type of information presented in each file is then validated through logic using the check results and instructs the way to process further the dataset. A Nucleotide blast and/or Protein blast is performed, respectively by extracting or translating the nucleotide sequences for each gene, and performing a blast search of each gene/protein sequence against the gene/protein sequences of other genomes. For each blasted gene/protein, the best score is kept during parsing of the results. The blastn/blastp files are parsed and joined onto the data extracted from the sequence in the parquet format. One file is created by blast type. The plot job creates a plot, colour-coded according to gene conservation, based on the computed matches and the file containing the sequence names and orientations. The plot is available to users in svg and png formats. The title, colour palette, shape, as well as other features are configurable. The full list of configurable options is available in the Readme.md (https://github.com/vestalisvirginis/synphage/blob/main/README.md) page of the repository.

### 2.3 Output files

Six different parquet files are generated during the transform step of the pipeline (Fig. 1A). The blastn summary.parquet and blastp_summary.parquet files (Fig. 1A) is a collection of records representing the best match for each locus/gene against each genome. The percentage identity between them is used to define the colour gradient of the cross-links between sequences when a match between adjacent sequences is present. The gebank_df.parquet and processed_genbank_df.parquet files (Fig. 1A) contain all the genes and coding sequence (CDS) feature information for all sequences processed through the pipeline. If the GenBank files contain only features of type CDS, the locus_tag id is replaced by the protein identifier protein_id. The gene_uniqueness.parquet and protein_uniqueness.parquet file combines both blastn_summary.parquet or blastp_summary.parquet and processed_genbank_df.parquet tables into one (Fig. 1A), allowing users to quickly filter for unique genes/proteins in their sequence of interest, or to quickly inquire about how many matches their genes/proteins of interest have retrieved, and in which other sequences, for instance.

The colour_table.parquet table is generated from the gene(or protein)_uniquenes.parquet table each time a new graphic is created. It contains the number of positive matches for each gene/protein within the plotted sequences, and it is used to attribute the colour for each gene/protein in the plot. The synteny plot is the graphical representation of the genome maps, and the colour code allows users to visualize conserved versus unique genes/proteins, linking gene/protein conservation levels to a coloured gradient-based figure (Fig. 1A).

### 2.4 Additional features

The synphage software allows users to directly download sequences from the NCBI database to be subsequently processed through the pipeline (Fig. 1A). This feature uses the Entrez Application Programming Interface (API) of the NCBI database, and a valid email address is required to use this functionality. The users’ API_key can also be passed as an environment variable. For more information about access to the NCBI database, see Entrez Help (https://www.ncbi.nlm.nih.gov/books/NBK3837/).

### 2.5 Limitations

Although data processing has been improved, the time required to run the pipeline, mainly the blast step, can be substantial when the number of sequences is large. We tested the software on a MacBook Pro (M1 chip) with 35 sequences: step_2 (validation) took 01:58.7 ± 00:11.5 (m:s); step_3a (blastn) took 12:21.0 ± 00:39.0 (m:s); step_3b (blastp) took 12:37.3 ± 00:18.0 (m:s); and step_4 (plot) took 00:07.3 ± 00:00.6 (m:s). Concerning downloading, this took 01:13.3 ± 00:00.6 (m:s) for the 35 GenBank files. The time required is mainly limited by the number of genomes requested and the database occupancy. However, the strength of synphage lies in its ability to generate an infinite number of plots with different sets of genomes, genome orientation and configuration, including diagram colours, without having to recompute the data.

## 3 synphage use cases

The utility and features of synphage are illustrated below.

### 3.1 Published versus unpublished sequences

With synphage, users can either transfer their own sequences into the GenBank folder or run the download job to retrieve sequences from the NCBI database using the search_key configuration. In the example illustrated (Fig. 1B–E) we used both functionalities. The downloaded sequences were obtained using the keywords: “Spbetavirus”[Organism] AND complete genome[Title] AND phage[Title] (Fig. 1C).

### 3.2 Visualizing unique versus conserved genes

Users can select the sequences they intend to visualize using a csv file. For our example (Fig. 1E) we selected 10 sequences of SPbetaviruses for plotting. The colour code allows us to visualize at a glance genes that are strictly conserved (purple), genes that are only partially conserved (blue to green) and genes that are unique (yellow) among the 10 sequences of relatively closely related genomes. In addition to the diagram, the table used to colour-code the plot is saved as a parquet file and can be easily queried to obtain the names of unique or strictly conserved genes. In Fig. 1C, we can see that the sequence MT601272 contains only six unique genes, and all are annotated as hypothetical.

### 3.3 Querying the users’ genes of interest

Users can also query genes of interest in the uniquenes.parquet table generated during the transform job. For instance, querying *yonO*, an RNA polymerase of SPbeta involved in the latter stages of infection (Forrest *et al.* 2017), shows that *yonO* is strictly conserved in our genomes of interest, and gives the corresponding locus_tag/gene in other genomes.

## 4 Conclusion

The synphage tool is intuitive, easy to use, and can generate genome diagrams and visualize gene conservation at a glance. It runs locally and offers a web interface to easily follow the data processing if required. It is primarily designed for phage research, although its use can be extended to plasmids or to specific loci or operons of bacterial genomes. Future work will include enhancing the user experience (UX), performance, and offering an interactive tool for more in-depth analysis, with the possibility to include additional data related to genes such as transcriptomic data and with the possibility to modify the plot layout and features.

## Data Availability

The data presented Fig. 1 and Section 3 are available in the Nucleotide NCBI database (https://www.ncbi.nlm.nih.gov/nucleotide/) and can be easily accessed using their name (see Fig. 1B). All the code used to generate the synteny diagram (Fig. 1E) and the tables used for the queries presented Fig. 1C and D is available on GitHub (https://github.com/vestalisvirginis/synphage). The query results can be reproduced using any Python libraries such as Pandas, Polars, DuckDB, or PySpark allowing it to work with parquet files or with free software such as Tad (https://www.tadviewer.com).
